# Lifelong effects of prenatal and early postnatal stress on the hippocampus, amygdala, and psychological states of Holocaust survivors

**DOI:** 10.1038/s41598-023-40618-3

**Published:** 2023-08-24

**Authors:** Monika Fňašková, Pavel Říha, Markéta Nečasová, Marek Preiss, Ivan Rektor

**Affiliations:** 1 Central European Institute of Technology (CEITEC), Brain and Mind Research Programme, Masaryk University, Brno, Czechia; 2 First Department of Neurology, St Anne’s University Hospital and Faculty of Medicine, Masaryk University, Brno, Czechia; 3 National Institute of Mental Health (Czechia), Prague, Czechia

**Keywords:** Neuroscience, Stress and resilience

## Abstract

This study focuses on hippocampal and amygdala volume, seed-based connectivity, and psychological traits of Holocaust survivors who experienced stress during prenatal and early postnatal development. We investigated people who lived in Central Europe during the Holocaust and who, as Jews, were in imminent danger. The group who experienced stress during their prenatal development and early postnatal (PreP) period (n = 11) were compared with a group who experienced Holocaust-related stress later in their lives: in late childhood, adolescence, and early adulthood (ChA) (n = 21). The results of volumetry analysis showed significantly lower volumes of both hippocampi and the right amygdala in the PreP group. Seed-based connectivity analysis revealed increased connectivity from the seed in the right amygdala to the middle and posterior cingulate cortex, caudate, and inferior left frontal operculum in the PreP group. Psychological testing found higher levels of traumatic stress symptoms (TCS-40) and lower levels of well-being (SOS-10) in the PreP group than in the ChA group. The results of our study demonstrate that extreme stress experienced during prenatal and early postnatal life has a profound lifelong impact on the hippocampus and amygdala and on several psychological characteristics.

## Introduction

War-related stress undeniably leaves marks on the minds and brains of survivors^1–3^. In the case of traumatic stress experienced during the prenatal period and in early childhood, stress affects the brain during this highly sensitive period and disrupt the process of normal development of brain structures and functions^4,5^. Developing structures and the connections between them can be damaged by high levels of cortisol, which is released due to chronic stress. Maternal prenatal stress causes an increase of cortisol and corticotrophin-releasing hormones (CRH) and the inhibition of the expression of the glucocorticoid barrier enzyme—11β-HSD2 in the foetus. CRH can influence the development of the hippocampus as well as of parahippocampal and limbic regions rich in CRH receptors^6^. Although cortisol is necessary for foetal growth and maturation, overabundant levels can lead to changes in neuronal development, including decreases in hippocampal volume. The hippocampus is a structure typically associated with learning and memory, and structural changes caused by stress are associated with anxiety, depression, and behavioural changes^7^. A decrease in hippocampal dendritic length^4^ and the reduction of the hippocampal volume as a consequence of prenatal stress have been described in animal studies^6,8^ and in a few human studies^9–11^. Next to the hippocampus, the amygdala is another limbic region susceptible to stress. The amygdala is involved in emotion processing and emotional responses and implicit memory; the amygdala and hippocampus are a basal structure of socioemotional functions^12^. Maternal anxiety and stress affect the form and function of the offspring’s amygdala^13,14^. Like prenatal stress, early postnatal stress disturbs the glucocorticoid and hypothalamic–pituitary–adrenal axis regulation and may lead to the disruption of glucocorticoid-sensitive areas later in life^15^. Changes in the amygdala and hippocampus as a consequence of stress in early life have been described in a review by Tottenham and Sheridan^16^; smaller volumes of these structures are linked to behavioural problems, and in the case of the hippocampus^17^, its reduction is associated with a risk of depression^18^.

In this study, we examine the lifelong impact of prenatal and early life stress in a unique group of Holocaust survivors. These participants were exposed to extreme stress throughout their prenatal development and subsequently in early childhood, when their pregnant mothers were in direct mortal danger. The cohort examined in this study is rare because very few Jewish people born during the Holocaust have survived and are alive more than 70 years after the Holocaust.

In relation to the impact of stress on the brain, the literature often mentions the prefrontal cortex, hippocampus, amygdala^19–22^, insula, and anterior cingulum cortex^23–25^. However, in our previous work examining the long-term effects of stress in Holocaust survivors^1^, we found no changes in hippocampal or amygdala volume; similarly, other MRI studies have found no difference in the hippocampus in Holocaust survivors^26^. Lupien et al. suggested that the brain structures susceptible to stress have sensitive periods called ‘windows of vulnerability’^5^. Other studies have indicated that the timing of the exposure of a stressor to the organism plays a key role in the subsequent effects on brain structures^9,14,16,17,17,27^. We suggest that for the hippocampus and amygdala, as limbic structures with socioemotional function sensitive to high cortisol release, the prenatal and early postnatal development is central to the subsequent formation of these areas. We also hypothesized that prenatal and early postnatal stress damages stress-sensitive structures and has lifelong effects that are detectable even after more than 70 years.

## Methods and materials

### Ethical consideration

The data were acquired in accordance with the Declaration of Helsinki. The Ethics Committee of Masaryk University approved the study. Prior to the examination, each participant reviewed and signed an informed consent form indicating whether they consented to secondary data processing.

### About research

In the present study, we examined the consequences of stress experienced in different age phases in Holocaust survivors, emphasising the prenatal period and early postnatal life in contrast to stress experienced in late childhood, adolescence, and young adulthood.

This study is part of a research project focused on the impact of stress on Holocaust survivors (HS) and two generations of their offspring. The investigation was conducted between 2015 and 2022 at the CEITEC Neuroscience Centre at Masaryk University in Brno, Czech Republic^1,28–31^. Volunteers were recruited through collaboration with local Jewish communities, announcements in the media and social media, on the university website, and through a snowball sampling method.

### Participant characteristics

#### Demographic information

All the participants were of Czech or Slovak origin (they lived in one country – Czechoslovakia, until its separation into two countries in 1993). During World War II, the participants and their parents were persecuted because of their Jewish origin.

Prenatal and early postnatal (PreP) group: Eleven participants (7 females, 64%; 4 males 36%) with a median age of 73 (72–77) years. Childhood, adolescence, and young adulthood (ChA) group: Twenty-one participants (15 females, 71%; 6 males 29%) with a median age of 84 (78–95) years.

To compensate for the different ages of the PreP and ChA groups in MRI data processing, a control group (CG) of non-Jewish participants with no Holocaust-related history was used. The data were not used for any other statistical comparisons; the CG was used only for the MRI analysis. Details are described in the chapter on data analysis. The CG consisted of 28 participants (17 females, 61%; 11 males, 39%) with a median age of 77 (70–86) years.

#### Life of the participants during the Holocaust

The situation in Czechoslovakia, where the survivors were born, was already strongly influenced by anti-Semitism after the Munich Contract in 1938 led to the partial occupation of Czechia; there was a radical increase in anti-Semitism after the German occupation of Czechia and the establishment of a Slovak fascist state in March 1939. The basic rights of the Jewish population were suppressed, and social and professional exclusion occurred. This was followed by the confiscation of property and homes, the separation of families, and deportations to ghettos and concentration camps. Most of the people who were deported were murdered. Some managed to avoid the transports but either had to live in hiding in poor conditions and in constant fear of discovery or later joined the partisans (for a deeper insight into the issue see: *The Holocaust explained—Czechoslovakia*^32^ or *In the Shadows of the Holocaust and Communism* about post-war life^33^).

The examined subjects were born during or shortly after the war, a time when their pregnant mothers were in direct danger of being murdered. In Czechia, all Jews were deported; some mothers of the participants in this study were in hiding. In Slovakia, some exceptions were made for physicians, pharmacologists etc. who were not deported as Slovakia did not have enough specialists in these fields; however after the insurrection of the Slovak army against the fascist regime in August 1944 all captured Jews were deported. Those who survived the atrocities of the Shoah often had no home after the war and experienced further trauma when they learned of the loss of their loved ones and the devastation of their community. This entire period lasted approximately 6–7 years.

##### PreP group

The median age of PreP participants in 1945 was 2 years (0–5). Depending on the age of the survivors, they had lived in hiding for 9 to 60 months. The PreP group (11 participants) mostly lived in hiding: in the homes of non-Jewish relatives or family friends (5 participants), in bunkers in the Slovak mountains (4 participants), and in a secret place (1 participant). One participant survived in the Terezín-Theresienstadt ghetto.

One participant was excluded from fMRI analysis due to the low quality of the data.

##### ChA group

The median age of the ChA participants in 1945 was 11 years old (6–24). Some of the ChA (21 participants) were forced to live in ghettos or were imprisoned in concentration, extermination, or forced labour camps at Auschwitz, Bergen-Belsen, Buchenwald, Dachau, and Mauthausen (11 participants); some lived in hiding (10 participants), mostly in the mountains, or they joined the partisan resistance.

### Participant excerpts

As examples of specific cases, memories of the Holocaust period from the perspectives of two probands of this study are briefly presented here. These testimonies were obtained from interviews that were part of the examination.

**Proband 1** (PreP group) was born in 1941 in a small town in the west of the Slovak state (present-day Slovakia). His father was a respected dentist, but because of their Jewish origin their family property was confiscated during the war and they were forced to move to a small underground apartment with poor conditions. Despite this, his father was able to continue his dental practice. After the Slovak National Uprising in 1944, one of his father’s patients warned the family of the danger of being transported to Auschwitz. They managed to escape. Within a few days, they reached the mountains where, with the help of some local people, they dug a bunker in which to hide. After some time, their hiding place was exposed and they had to hide in another place. The family managed to survive hiding in the mountains for nine months thanks to a few local people who brought them food secretly.

After their liberation, the then four-year-old proband was afraid to speak: *For nine months in hiding, I had to keep my voice down so as not to reveal us. After the liberation I was unable to speak out loud, and for some time after my return to normal life I only whispered.*

As an adult, he felt guilty that he had managed to survive while others had not*: I had nightmares. I could have turned to ash like others who were not as lucky as I was.*

**Proband 2** (ChA group) was born in 1928 and lived with his family in a South Moravian town in Czechoslovakia until 1938, when the town was annexed by Nazi Germany. After this, the family moved to the nearby city of Brno, where he attended gymnasium—the last Jewish gymnasium in the country, as all other Jewish grammar schools had been banned. Describing the situation after the war, he said, *Of the twenty boys, I was the only one left alive; of the eighteen girls, three returned*.

In April 1943, he and his older sibling were transported from Brno to the ghetto in Theresienstadt, where he initially did digging work. Later, he worked in a shoemaker’s workshop. By fixing the shoes of people in the ghetto after work, he earned a little money for food: *And that’s why I looked quite good in front of Dr Mengele*.

From Theresienstadt, both siblings were transported to Auschwitz in September 1944: *High up in the dark sky there were flames. The chimneys were not visible. No one knew that they were flames from the crematorium furnaces.* He and his sibling also passed Mengele’s selection because they claimed to be slightly older than they actually were. They were transferred from Auschwitz to the Landsberg concentration camp for work after a week.

At Landsberg, they did very hard work: *It was an extermination camp, horrible camp. We pulled logs from the forest or worked in underground factories, and on the way back we carried the dead who hadn’t survived the work for several kilometres*. His sibling also did not survive the camp and died in February 1945.

After three months, in December 1944, the proband was transferred to Landshut camp, where he worked in atrocious conditions in freezing temperatures. He became seriously ill. The Germans closed Landshut in the spring of 1945 and evacuated the prisoners to Dachau. He was liberated in April 1945 in the hospital of Dachau concentration camp, where he was on the verge of death.

*For fifty years I was not able to talk about my experiences*, stated this proband.

### Exclusion criteria

Subjects with a history of massive brain trauma, stroke, tumour, progressive brain disease, cognitive decline, or systemic illness or with contraindications for MRI (metal implants, pacemaker, or claustrophobia) were not included in the study.

### Instruments

#### Initial screening

All participants underwent a 7-min screen test^34^ (7MS), Mini-Mental State Examination^35^ (MMSE; cut-off 26 points), and the Geriatric depression scale test (GDS-15; cut-off 6 points)^36^ as an initial screening. No individuals with symptoms of moderate or severe depression or cognitive decline participated in the study.

#### Psychological questionnaires

Participants were examined for persistent symptoms of stress using four psychological questionnaires. The Trauma Symptoms Checklist (TSC-40)^37^ is a 40-item instrument assessing symptoms of traumatic stress; the PTSD Checklist—Civilian Version (PCL-C)^38^ is a 17-item questionnaire for screening of posttraumatic stress and evaluating posttraumatic stress symptoms. The Post Traumatic Growth Inventory (PTGI)^39^ is a 21-item questionnaire that measures potential posttraumatic growth as a positive legacy of trauma. Well-being was investigated with the Schwartz Outcome Scale-10 (SOS-10)^25,40^.

#### MRI

##### Data acquisition

MR examinations were performed on a 3 T scanner Siemens Prisma using a 64-channel head coil. The MRI protocol included 3D T1-weighted magnetisation prepared rapid gradient echo (MPRAGE) sequence with TR = 2.3 s, TE = 2.33 ms, TI = 0.9 s, FA = 8°, isometric voxel size 1 mm in FOV 224 × 224 mm and 240 slices and 3D fluid-attenuated inversion recovery (FLAIR) sequence with TR 6000 ms, TE 387 ms, TI1 1900 ms, vox 1 × 1 × 1 ×  mm.

Functional protocol used T2 echo-planar imaging sequence with TR = 2.51 s, TE = 35 ms, flip angle = 70, isometric voxel size 3 mm in FOV 192 × 192 mm and 44 slices. The number of scans was 200. All subjects were instructed to close their eyes while not falling asleep during the resting state.

Some data were obtained at a partner site, NÚDZ Klecany, with the same type of 3 T Prisma scanner, multichannel coil, and protocol sequence.

Data from all participants were manually checked for artefacts; pathology was checked by an experienced radiologist. Participants who did not meet our quality criteria and scans with technical artefacts were excluded from the study. We excluded two participants, one from each group.

### Data analysis

#### Hippocampus and amygdala volumetry

Anatomical MRI data were segmented and parcellated using Freesurfer 7.1.1 and the Desikan–Killiany cortical atlas^41^.

Prior to statistical analysis, we corrected hippocampal and amygdala volumes for effects of no interest, specifically brain size, age, sex, and MR scanner. First, volumes were multiplicatively corrected for total intracranial volumes using estimated total intracranial volume (eTIV). Second, the effects of sex age, and MR scanner were estimated and compensated using a general linear model (GLM). Because the effect of interest (stress-induced atrophy) and the effect of no interest (age-induced atrophy) interact in the target groups, we used an independent age-matched control group (28 participants with no Holocaust-related history scanned with same MR protocol) to estimate the effect of no interest. Estimated effects of age and sex were subtracted from the target groups (PreP and ChA).

**Figure 1:**
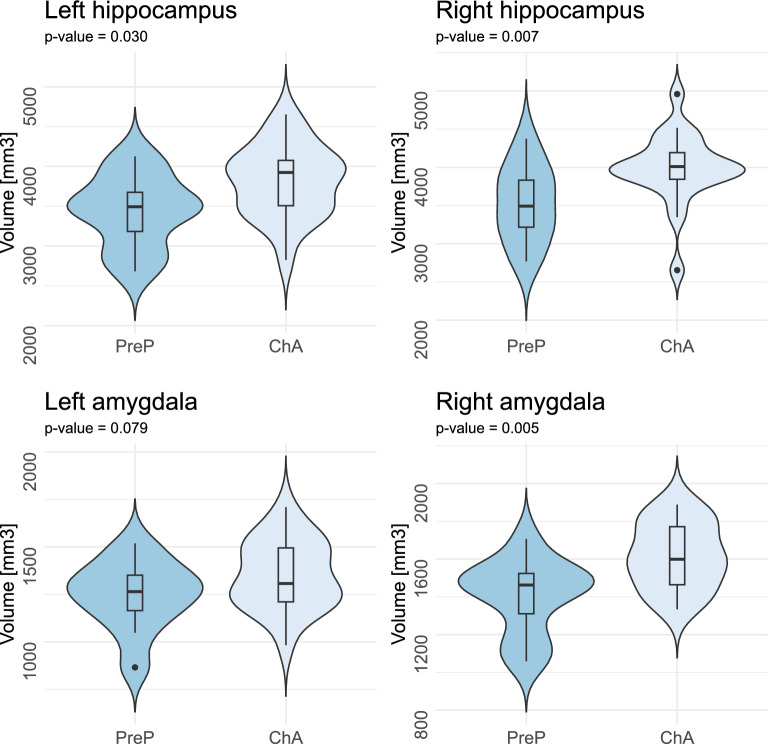
Volume differences. Legend: *Volume differences between PreP* (*N* = 11) *and ChA* (*N* = 21) *groups in the left hippocampus, right hippocampus, left amygdala, and right amygdala*. The p-value expressing the statistical difference between groups is given in the name of each subplot.

The aim of the study, to assess the differences between PreP and ChA groups in hippocampal and amygdala volumes, was achieved using a one-sided non-parametric Mann–Whitney rank test. A nonparametric test was used because the data in our small sample did not meet the requirements of parametric tests, i.e., the data did not have a normal distribution. The histogram in the violin plots (Fig. 1) shows the distribution of the data.

#### Functional data processing

Data were processed in Matlab and SPM12. Preprocessing consisted of realign (motion correction), coregistration of anatomical scans to BOLD data, spatial normalisation using MNI template, and spatial smoothing using gaussian kernel with FWHM of 6 mm. Subsequently, very low frequencies were removed from the BOLD data using a high-pass filter with a cut-off of 128 s, and other nuisance effects were regressed out of the BOLD data—specifically white matter and CSF signals and 6 movement parameters obtained from the realign procedure.

Resting-state fMRI were analysed using seed-based connectivity maps from the left and right hippocampus and amygdala. Individual connectivity was calculated based on correlations between the ROI signal (first principal component) and all other voxels in brain.

Group statistics were calculated with a second-level model using SPM12. A two-sample t-test comparison of individual connectivity maps of the PreP group and ChA group was performed. Sex and age variables were corrected using the control group in the same way as for volumetry.

## Results

The PreP and ChA groups were not significantly different in sex and education (two-sided Fisher’s exact test; sex *p* = 0.7, education *p* = 0.8).

Depression symptoms were experienced by 3 PreP (27.3%) and by 5 ChA (23.8%). There was not a significant difference between PreP and ChA (*p* = 0.5).

### Psychological questionnaires

The results of psychological testing between the PreP and ChA groups (Table 1) were significantly different for the TSC-40 (*p*—0.01) and SOS-10 (*p*—0.03) questionnaires. The PreP group had higher scores in traumatic stress (TSC-40 questionnaire) and lower scores in well-being (SOS-10).

**Table 1:** Psychological questionnaires—differences between PreP and ChA.

Measure	PreP			ChA			*p*
	Median	Mean	SD	Median	Mean	SD	
TSC-40	29	23.1	12.1	15	13.0	8.9	**0.01**
PCL-C	38	35.8	10.0	30	30.5	9.3	0.11
PTGI	62	63.3	15.9	55	56.5	20.4	0.37
SOS-10	46	42.0	10.3	50	49.5	6.2	**0.03**

*TSC* trauma symptom checklist, *PCLC* C: PTSD checklist—civilian version, *PTGI* posttraumatic growth inventory, *SOS* Schwartz outcome scale.

Significant values are in bold.

### MRI

A significantly smaller GM volume was observed in the right (*p* = 0.004) and left (*p* = 0.025) hippocampus and right amygdala (*p* = 0.004) in the PreP group as compared to the ChA group. The volume of the left amygdala was reduced, but the statistical value did not reach significance (*p* = 0.054) (Fig. 1).

### fMRI

**Figure 2:**
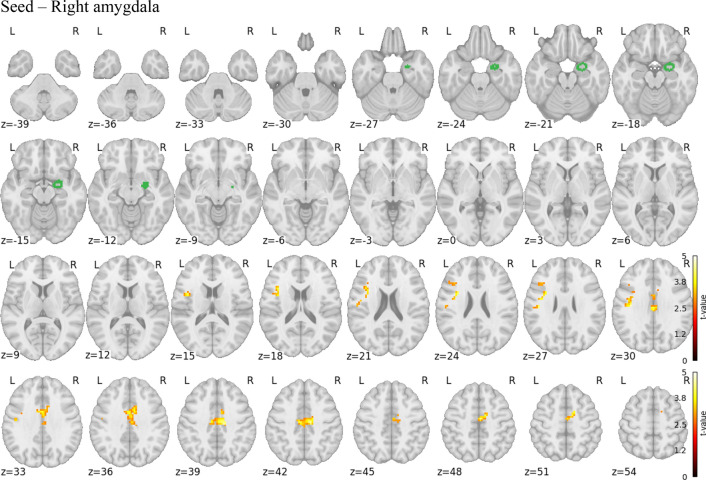
Seed-based connectivity. Increased connectivity from seed in right amygdala to middle and posterior cingulate cortex (bilateral), caudate (bilateral), and frontal operculum inferior (left).

For the seed-based connectivity analysis, we examined seeds in the right and left hippocampi and amygdalae. Seeds from right amygdala revealed significant increased connectivity in the PreP group in contrast to the ChA group. Seed based in the right amygdala showed increased connectivity to these areas: bilateral middle and posterior cingular cortex and frontal operculum inferior (Fig. 2).

## Discussion

The participants in this study were exposed to an extremely stressful environment during their entire intrauterine development and early postnatal life. More than seven decades later, the signs of stress are still evident. The PreP group showed significantly higher levels of traumatic stress and lower levels of well-being compared to the Holocaust survivors who were born before the Holocaust—the childhood, adolescence, and young adult (ChA) group. MRI results show reduced volumes of both hippocampi and the right amygdala (the left amygdala was also reduced but did not reach a significant difference) and fMRI results revealed increased connectivity the right amygdala to the medial and posterior cingulum bilaterally and gyrus frontalis inferior in the PreP group as compared to the ChA group.

The data from this study indicates that PreP stress may have at least a similar or greater lifelong impact than extreme stress perceived by children and young adults. This can be explained by the fact that the developing brain (in the PreP group) is more damaged than the more developed brain (in the ChA group), although those who experienced prenatal stress and early postnatal stress (PreP) were not consciously aware of this risk as they were exposed to stress indirectly through their mother.

Pregnancy was not common among Jewish women during the Holocaust and made the pregnant women even more vulnerable to the threats faced as members of a persecuted ethnic group^42^. Stress transmitted from mother to foetus affects the brain during a very sensitive period when brain structures are developing and growing. The hippocampus, like other structures, continues to develop after birth; Lupien stated that hippocampal development and maturation after birth is most rapid in the first two years of life^5,43^. Disruption of hippocampal development during this period affects its overall shape and may alter its function in later life. The median age of the PreP group in the current study was 2 years in 1945, which means the stress exposure lasted throughout the main hippocampal developmental stage. The amygdala develops in the early stages of prenatal life and, like the hippocampus, is very sensitive to the effects of glucocorticoids^44^. The association between the amygdala and anxiety is already evident in infants. Fear of strangers and fear of heights, which appear in children at the end of the first year of life, indicate the maturing of the amygdala’s role in fear response^45^. An adverse environment in the first months of life after birth plays an important role that affects the developing amygdala later in life^12^. Studies focusing on the effect of prenatal and early postnatal stress on the amygdala have not shown consistent results: both increases and decreases in volume were found^14,46–50^. A non-linear alteration of the amygdala volume after early-life adversity was reported^17^. Our results show a persistent reduction in amygdala volume 70–75 years after prenatal and early postnatal stress. Our findings suggest that the timing of stress exposure is a major cause of grey matter changes.

The crucial influence of stress in early human development is supported by our results on the functional connectivity of the brain, where we found right amygdala in the PreP group as compared to the ChA group.

Seed based analysis in the right amygdala show increase connectivity to the opercular part of the inferior frontal gyrus, middle and posterior cingulum. In our previous work^1^, in which survivors were compared with a control group, we found, among other things, a reduced volume of the anterior but not middle or posterior cingulum. Similar to this research, a study of the effects of social stress revealed increased connectivity between the amygdala and posterior cingulum in participants exposed to stress^51^. The volume of the pars opercularis in the frontal lobe has been found in patients with major depressive disorder^52^. The higher connectivity to these structures shown by PreP compared to ChA shows that stress during prenatal development and soon after birth modifies brain function in the long term.

Regarding the lifelong effects of early stress on the psyche, we can compare our results with a study conducted immediately after the war by Anna Freud and Sophie Dann^53^. They examined the psychological state of children born during the war whose parents were deported to concentration camps and killed. These children spent several years in Terezín-Theresienstadt and came to England after the war. The children showed persistent anxiety, distrust, and aggression towards their new caregivers. These children found it difficult to establish relationships with adults, and several attempts at adoption ended in failure. Over time, they adapted. Freud and Dann described the effects of PreP stress in children shortly after exposure to a traumatic event. Our results extend this study in terms of long-term consequences, where we see that the effects of stress are evident even at older ages, 70–75 years after the war.

Our previous study^1^ demonstrated the lifelong impact of extreme stress caused by the Holocaust on stress-related psychological features and on brain structures (insula, anterior cingulate, medial prefrontal cortex, etc.); however, the hippocampus and amygdala volume were not significantly reduced. The present study confirms our hypothesis, showing a smaller volume of the hippocampus and amygdala in the PreP stress group as compared to the ChA stress group.

## Conclusion

Extreme PreP stress has an profound lifelong impact on the brain. The present study displays lifelong changes in stress-related psychological features and structural brain changes detectable after more than 70 years. Our findings support the importance of the prenatal period and early postnatal development on socioemotional structures—the hippocampus and amygdala. It confirms that the extreme stress-related reduction of the volumes of the hippocampus and amygdala is age-dependent, as it occurs in persons exposed to extreme stress in PreP but not in later life periods. To the best of our knowledge, this is the first (and probably the last) study to examine the effect of prenatal and early postnatal stress on the hippocampus and amygdala in Holocaust survivors. The results of our study serve as a contribution to a hitherto understudied area of neuroscience dealing with the lifelong consequences of early war-related stress, with a focus on limbic structures.

## Limitations

The main limitation of this study is the small sample size due to the age and characteristics of the participants, because Jewish children born during the war usually did not survive the war, so our PreP group is small but unique. Further, we cannot accurately assess whether the smaller volumes of brain structures in the PreP group were due directly to the stress they experienced during the Holocaust or whether this stress made them more vulnerable to other stresses and traumatic events that they experienced later in life. Unfortunately, we do not have detailed information on the health status of our participants’ mothers during pregnancy.

## Data Availability

The datasets used and/or analysed during the current study available from the corresponding author on reasonable request. The authors assert that all procedures contributing to this work comply with the ethical standards of the relevant national and institutional committees on human experimentation and with the Helsinki Declaration of 1975, as revised in 2008.

## Abbreviations

CRH: Corticotropin-releasing hormone
GDS: Geriatric depression scale
GM: Grey matter
HS: Holocaust survivors
ChA: Childhood, adolescence, adulthood stress
MRI: Magnetic resonance imaging
PCL-C: Post-traumatic stress disorder checklist—civilian version
PreP: Prenatal and early postnatal
PTGI: Post-traumatic growth inventory
PTSD: Post-traumatic stress disorder
SOS-10: Schwartz outcome scale-10
TSC-40: Trauma symptoms checklist (TSC-40)
